# P-4. Real-world Impact of Quadrivalent Human papillomavuris vaccination on Genital Warts and High-grade Cervical Intraepitelial Neoplasia Hospitalization rates in Brazil

**DOI:** 10.1093/ofid/ofae631.215

**Published:** 2025-01-29

**Authors:** Juan Carlos Orengo, Bruna Cristina Lima, Cintia I Parellada, Tulio Tadeu Rocha sarmento, Julio Oliveira Barbour, Rodrigo Gonçalves Queijo, Ana Luiza Bierrenbach

**Affiliations:** MSD (IA) LLC, Guaynabo, Puerto Rico; MSD Brazil, São, Sao Paulo, Brazil; MSD Brazil, São, Sao Paulo, Brazil; Precision Data, São pa, Sao Paulo, Brazil; Precision Data, São pa, Sao Paulo, Brazil; Precision Data, São pa, Sao Paulo, Brazil; Hospital Sirio Libanês, São Paulo, Sao Paulo, Brazil

## Abstract

**Background:**

In Brazil, the quadrivalent HPV vaccination (4vHPV) was introduced into the National Immunization Program (NIP) in 2014 for girls aged 9-13 years old and later expanded to include boys aged 11-13 years old in 2017. The vaccination coverage rate for ≥ 1 dose was 76.1% for females and 49.1% for males in 2019. There is a lack of published data on the clinical impact of HPV vaccination in Latin America. A public hospital database, that covers approximately 75% of Brazilian population, offers an excellent opportunity to generate real-world evidence.

**Figure 1.**
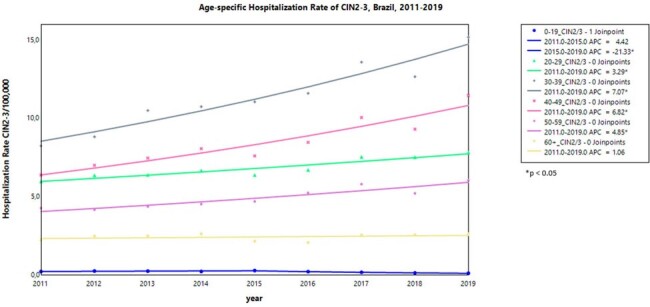
Hospitalization rate trends of High-grade Cervical Intraepithelial Neoplasia, Brazil, 2011-2019

**Methods:**

This was a time-series study to evaluate the impact of the 4vHPV program on genital warts (GW) and high-grade cervical intraepithelial neoplasia (CIN2/3) hospitalization rates in Brazil from 2011-2019. The number of cases were obtained using ICD-10 codes and age-specific hospitalization rates per 100,000 people were calculated. Annual percent changes (APC) in hospitalization rates were assessed by joinpoint regression (statistical significance: p< 0.05).

**Figure 2.**
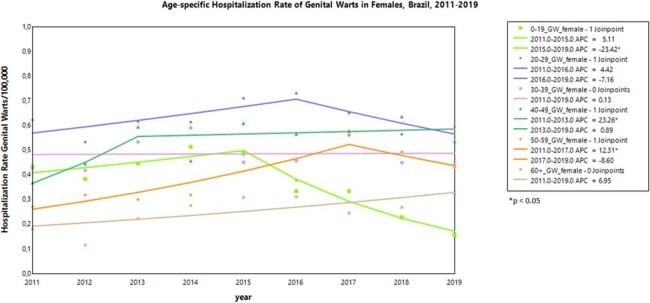
Hospitalization Rate trends of Genital Warts in Females, Brazil, 2011-2019

**Results:**

From 2011-2019, there were a total of 47,500 hospitalizations for CIN2/3 and 11,230 hospitalizations for GW (4,135 in women and 7,095 in men). In females aged < 20 years, there was a significant downward trend in CIN2/3 hospitalizations, with an annual rate decrease of 21.3% from 2015-2019. Other age groups showed a significant increasing trend during the study period, except for those aged ≥ 60 years (Fig 1). GW hospitalization rates in females aged < 20 years showed a significant downward trend of 23.4% per year from 2015-2019, while remained stable for other age groups (Fig 2). Among males, GW hospitalization rates showed an increasing trend in most age groups from 2011 to 2015. However, starting from 2015, there was a significant decrease in males< 20 years at a rate of per year 10.8%, age groups of 20-29 years and ≥60 years showed non-significant reduction changes (Fig 3).

**Figure 3.**
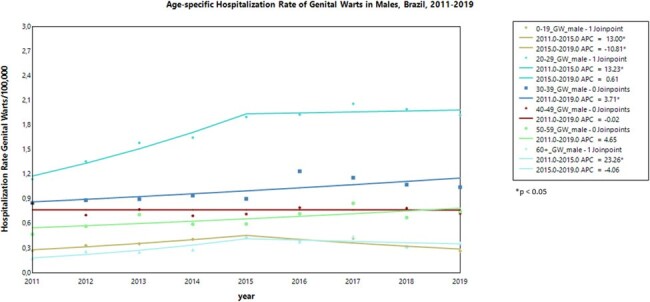
Hospitalization Rate trends of Genital Warts in Males, Brazil, 2011-2019

**Conclusion:**

Using a hospital nationwide database, this study provides evidence of the early impact in the 4vHPV on GW and CIN2/3 at the population level in targeted cohorts of females and males as well as non-targeted cohorts of males in Brazil. High vaccination coverage in females and males as well as expansion for other age groups are critical to continue seeing the reduction of HPV-associated diseases in Brazil.

**Disclosures:**

**Juan Carlos Orengo, MD, MPH, PhD**, Merck & Co., Inc: Employee|Merck & Co., Inc: Stocks/Bonds (Private Company) **Bruna Cristina Lima, n/a**, Merck & Co., Inc: Employee **Cintia I. Parellada, MD, PhD**, Merck & Co., Inc: Employee|Merck & Co., Inc: Stocks/Bonds (Private Company) **Tulio Tadeu Rocha sarmento, MSc**, Precision data: Employee **Julio Oliveira Barbour, BSc, MD**, Precision Data: CEO|Precision Data: Stocks/Bonds (Private Company) **Rodrigo Gonçalves Queijo, BSc**, Precision Data: Employee **Ana Luiza Bierrenbach, MD, PhD**, Precision Data: Consultant

